# 

**Published:** 1892-03-05

**Authors:** 


PRICE ONE PENNY.

o. 284, Vol. XI.—March 5th, 1892.

egistered at the General Post Office as a Newspaper for ransmission in the United Kingdom and Abroad.]

WITH EXTRA NURSING SUPPLEMENT.
Per Annum, 6s. 6d., post free.
Monthly Parts, 6d.; post free, 8d.
Ditto per Annum, 8s., post free

### THE HOSPITAL

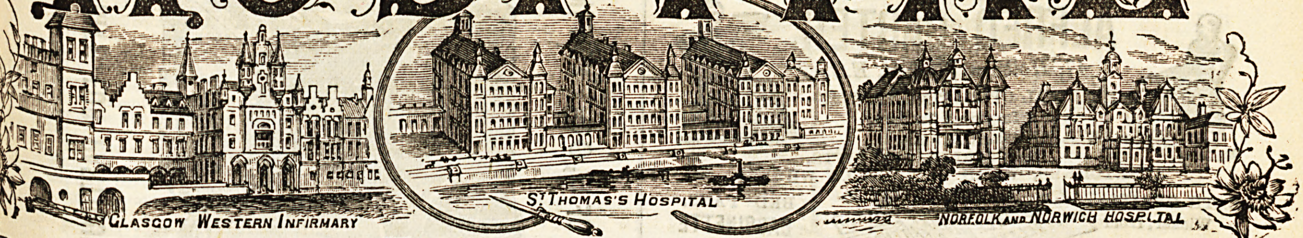

A WEEKLY JOURNAL OF

### Science, Medicine, Nursing, and Philanthropy.

SATURDAY, MARCH 5th, 1892.

**Overlapping** - 273
**Passing Topics.**—Charity—Limited. - 274
**The Doctor's Armchair.**—Payn on Physic - 275
**Some American Hospitals.** X —The Massachusetts General Hospital, Boston - 276
**Everybody's Column** - 277
**To Our Readers** - 278
**Notes and News** - 278
**General Charities—Scraps and Gleanings** - 279
**Annotations.**— Opium — Criminal Anthropology — Unhappy Shop Assistants - 280
**The Practitioner's Mirror and Retrospect—**
MEDICINE.—The Physiology of Œdema - 281
SURGERY.—The Surgical Aspect of Abdominal Tuberculosis 282
OPHTHALMOLOGY.—Foreign Bodies in the Conjunctival Sac - 283
**Some Scientific Aspects of Insanity.** XV.—Secondary Dementia (*continued*) - 284
**The Student of Medicine.**—Appointments—Vacancies

EXTRA SUPPLEMENT.—THE NURSING MIRROR.

**En Passant.**—Why Hospitals are Poor—Poor-Law Nurses—Clean Hands—Short Items—Amongst the Lepers - cxxxiii

**On the Nursing of Children.** IV.—Operation Cases (*continued*) - cxxxiv

**Notes and Queries** - cxxxiv

**For Reading to the Sick.**—Great Thoughts - cxxxv

**Notes from Australia** - cxxxv

**Wants and Workers** - cxxxvi

**The Queen's Nurses** - cxxxvi

**Everybody's Opinion.**—A Nursing Reserve - cxxxvii

**Nursing Uniforms—I.** Her Majesty's Nursing Sisters - cxxxvii

**Death in our Ranks—Appointments** - cxxxvii

**Off Duty.**—Betty's Angel - cxxxviii

**East London Nurses** - cxxxviii

